# Financial Literacy, Financial Education, and Smoking Behavior: Evidence From Japan

**DOI:** 10.3389/fpubh.2020.612976

**Published:** 2021-01-15

**Authors:** Somtip Watanapongvanich, Mostafa Saidur Rahim Khan, Pongpat Putthinun, Shunsuke Ono, Yoshihiko Kadoya

**Affiliations:** School of Economics, Hiroshima University, Hiroshima, Japan

**Keywords:** smoking, financial literacy, financial education, Japan, healthcare decision

## Abstract

In this study, we examine the relationship between financial literacy, financial education, and smoking behavior among the Japanese population. We hypothesize that financially literate and financially educated people, who have the ability to make more rational decisions, are less likely to smoke. Using the Preference Parameters Study of Osaka University, conducted in 2010 (*N* = 3,706), the probit regression results show that both financial literacy (with an emphasis on knowledge of investments) and financial education (with an emphasis on savings behavior) have a significant negative impact on smoking behavior. In addition, gender, age, education, marital status, household income and assets, risky behaviors, a myopic view of the future, risk preference, and level of happiness also significantly predict the likelihood of a person being a current smoker. This study provides empirical evidence that enhancing the rational decision-making ability of individuals through financial literacy and financial education may curtail smoking behavior.

## Introduction

Smoking has become an increasing public health concern in Japan over the past several decades. In 2012, the Japanese government set a target to reduce the smoking rate among adults aged 20 and over to 12% by 2022 (1). Since then, government and non-government organizations have taken several actions to curtail smoking, such as prohibiting indoor smoking, increasing the tobacco tax, and providing smoking cessation services (2). However, the prevalence of smoking in Japan has remained unchanged since 2013 (3–5). According to the Global Burden of Disease Study (GBD), Japan had the world's seventh-largest population of smokers in 2015 (6), and the 2017–2019 WHO report on the global tobacco epidemic indicates that 19% of Japanese aged 15 and over, ~21 million people, still smoke (4, 5). These statistics raise the question of why smoking is still relatively prevalent in the country despite continued efforts to curtail it. It also seems likely that the government will not reach its smoking reduction target in the near future.

Smoking has substantially negative health [e.g., (7–13)], behavioral [e.g., (14–16)], and economic consequences [e.g., (17, 18)]. One important question is why do many people still engage in smoking despite its harmful effects. Smoking, as an addictive behavior, could be explained from three competing theoretical frameworks: rational, imperfect rational, and irrational choice (19, 20). Becker and Murphy (21) advanced the rational choice theory and postulated that smoking is a rational choice made on the basis of utility maximizing criteria. This means that people, by using available information, find that present pleasure of smoking provides more utility than the utility loss from its adverse consequences. However, the rational choice theory has been challenged on the ground that smokers do not have all the relevant information at the time when they make smoking decisions, and they cannot objectively assess the value of information. We support the argument that smoking decisions are not rational, but rather either imperfectly rational or completely irrational.

Imperfectly rational framework postulates that smokers show bounded rationality because of their poor judgment over future health effects and time-inconsistent preferences. Compared with rational decision makers' time consistency over discounting immediate present and distant future outcomes, imperfectly rational smokers tend to be hyperbolic in discounting future consequences over current pleasure from smoking. Barlow et al. (22), MacKillop et al. (23), Ida and Goto (24), Baker et al. (21), and Mitchell (25) argued that smoking is associated with a high discount rate, meaning that smokers greatly discounted the future compared with the present outcomes. Barlow et al. (22) reviewed 69 relevant studies and argued that higher discounting was the reason for decisions to smoke and reluctance to quit even though smokers know future health consequences. O'Donoghue and Rabin (26) and Gruber and Köszegi (27) considered time-inconsistent people as biased toward the present. In other words, smokers value the pleasure they receive from smoking in the present whilst heavily discounting the negative consequences of smoking in the future (20). Besides hyperbolic discounting, several studies modeled time-inconsistent addictive behavior by the exponentiated hyperbolic discounting (28) and subadditive discounting (29, 30). In addition to time-inconsistency, smokers, particularly young smokers, are often found to have imperfect information; they lack judgment in understanding future health consequences and sometimes become addicted suddenly (31–35). The human capital model developed by Grossman (36) confirmed that smokers tend to place less emphasis on human capital as they heavily discount future health effects. Grossman (36) found that smoking destroys health capital, thereby decreasing human capital and ultimately taking a toll on productivity. Moreover, smokers often fail to recognize that the adverse effects of smoking not only affect them directly but also result in higher social costs from negative externalities and health care burdens (17, 37) compared with the social benefits of socially desirable outcomes (17). Cognitive limitation could be a possible reason why imperfectly rational people are unable to properly value the health consequences of smoking.

Finally, the conceptual framework that conjectures smoking as an irrational behavior postulates that decisions to smoke are guided by emotions, which hinder objective assessment of risks and benefits associated with the consumption. When emotions drive decisions, people lose their rationality and deviate from the objective assessment of information. Decision to smoke as an addictive good is sometimes triggered by external causes leading to impulsive behavior (20, 38). Moreover, Perikleous et al. (39) confirmed the evidence of peer influence on adolescent smoking, which confirmed that smokers do not always make unbiased decisions.

How to reduce people's tendency to smoke has been an area of great concern over the decades. One approach to prevent people from making imperfectly rational or irrational decisions is to enable them to make rational decisions. In this study, we propose that financial literacy is a rational decision-making instrument, as the people who possess this knowledge tend to behave more rationally; this is reflected in their economic and financial behavior (40–50). Moreover, financial literacy is related to improved cognitive ability (51, 52), which helps them become time-consistent in making judgment over current and future outcomes. Because of rational decision-making ability and increased cognitive ability, financially literate people are likely to make informed decision, place due importance on future outcomes, be time-consistent, and not be influenced by emotions. Thus, financially literate people, being rational agents, are less likely to be smokers because of their ability to objectively assess the benefits and health risks of smoking. In support of the ability to make rational decisions, previous studies provided evidence that financially literate people are engaged in better healthcare decision-making and health-promoting activities (53, 54). Because of the relevance of financial literacy with positive health behavior, better health outcomes, and quality of life in later years, positive reinforcement should be solicited in the society. Similar to financial literacy, financial education received in school is likely to make people aware of financial issues and help them to make informed and rational decisions (55–58).

Although previous studies show that financial literacy is associated with better health-related decision-making, few empirical studies have investigated the relationship between financial literacy and health behaviors. A recent study by Watanapongvanich et al. (59) used financial literacy and financial education to explain gambling behavior among the Japanese population. They found that financial literacy has a significantly negative relationship with gambling frequency, whilst financial education has an insignificant impact. Existing research also shows that the thought processes behind smoking and gambling have the same intertemporal relationship with the financial decision-making process (21, 25, 60, 61) and that gambling and smoking are proxies for irrational behaviors that can lead to negative consequences. Therefore, apart from gambling, it is worth investigating how financial literacy and financial education can reduce smoking behavior in Japan.

In this study, we examine the relationship between financial literacy, financial education, and smoking behavior. We hypothesize that financially literate and financially educated people, who have the ability to make more rational decisions, are less likely to be smokers. To the best of our knowledge, no existing empirical research addresses the association between financial literacy, financial education, and smoking behavior. Our study contributes to the existing literature by providing empirical evidence on the connection between financial literacy and financial education as a rational decision-making tool and how they affect irrational decisions tied to smoking behavior in Japan. The results of this study can help policymakers implement effective interventions to prevent and minimize the negative consequences of smoking.

The remainder of this paper is organized as follows. In sections Data and Methodology, the data and methodology are described, respectively. In section Results, the empirical results are summarized, in section Robustness Check, robustness of the results are checked, and in section Discussion, the results are discussed. Section Conclusion presents the conclusion.

## Data

### Data

In this study, we use data from the Preference Parameters Study (PPS) conducted by the Institute of Social and Economic Research at Osaka University. The PPS is a panel survey that collects information on socioeconomic characteristics and preferences from a representative sample of the Japanese population. In this study, we utilized data from the 2010 wave, which contained questions about smoking behavior, financial literacy, and financial education. The sample includes data from 3,706 individuals, or ~69% of all respondents in 2010 (5,386 individuals). We excluded individuals with missing data on smoking behavior, financial literacy, financial education, and demographic variables (1,680 individuals).

### Variable Definitions

The dependent variable in this study is smoking behavior. The PPS contains the question “Do you smoke?” and provides seven responses, where 1 means “do not smoke at all,” 2 means “hardly smoke,” 3 means “smoke sometimes,” 4 means “about 10 cigarettes a day,” 5 means “about a pack a day,” 6 means “more than two packs a day,” and 7 means “I used to smoke but have quit.” We grouped these responses into a binary scale of non-smokers and current smokers by coding respondents who answered 1, 2, or 7 as 0 or non-smokers (62) and those who answered 3, 4, 5, or 6 as 1 or current smokers.

There are two main variables of interest in our study: financial literacy and financial education. To measure financial literacy, we followed the methodology proposed by Lusardi and Mitchell (63), which is simple and widely adopted in existing literature [e.g., (59, 64–71)]. It uses the following three questions.

a. Suppose you had 10,000 JPY in a savings account and the interest rate is 2% per year and you never withdraw money or interest payments. After 5 years, how much would you have in this account in total?

- More than 10,200 JPY (correct answer)
- Exactly 10,200 JPY
- Less than 10,200 JPY
- Do not know
- Refuse to answer

b. Imagine that the interest rate on your savings account was 1% per year and inflation was 2% per year. After 1 year, how much would you be able to buy with the money in this account?

- More than today
- Exactly the same
- Less than today (correct answer)
- Do not know
- Refuse to answer

c. Please indicate whether the following statement is true or false. “Buying a company stock usually provides a safer return than a stock mutual fund.”

- True
- False (correct answer)
- Do not know
- Refuse to answer

The first two questions measure the respondent's understanding of how compound interest works and the effect of inflation. Indeed, the questions help evaluate a respondent's understanding of economic concepts and basic numeracy (63). The third question evaluates respondents' understanding of the concept of risk diversification. In this study, we assigned a score of one for each correct answer and 0 for each incorrect answer. We obtained the financial literacy variable by taking the equally weighted average scores of the three questions.

For financial education, the respondents were asked, “Did you receive any compulsory financial education when you were in elementary school?” with three possible responses: yes, no, and do not know. We coded the respondents who answered yes as one and those who answered no or do not know as 0. We treated this as a binary variable.

We should note an important difference between financial literacy and financial education in Japan. A recent work by Watanapongvanich et al. (59) suggests that Lusardi and Mitchell's (63) financial literacy questions measure respondents' current level of financial knowledge from an investment perspective. However, the Japanese school curriculum includes financial education to teach students about savings using a children's bank campaign (59, 72). Therefore, financial literacy that focuses on investment concepts is somewhat different from financial education that focuses on savings behavior (59). Sekita (71), who found that receiving financial education through a children's bank campaign has no effect on the level of financial literacy among representative Japanese adults, also supports this claim. Consequently, we include both financial literacy and financial education as explanatory variables in this study.

Furthermore, we include gender, age, university degree, marital status, household members, employment status, household income, and household assets as demographic variables in our specifications. We also control for risky behaviors (exercise, drinking alcohol, and gambling behavior), myopic view of the future, risk preference, level of happiness, and anxiety about health. Table 1 provides the definitions of all the variables.

**Table 1:** Variable definitions.

**Variables**	**Definitions**
Smoking behavior	Binary variable: 1 = current smoker (sometimes–more than two packs a day) and 0 = non-smokers (do not smoke at all, quit, or hardly smoke)
Financial literacy	Continuous variable: number of correct answers from three financial literacy questions
Financial education	Binary variable: 1 = received compulsory financial education at school and 0 = otherwise
Male	Binary variable: 1 = male and 0 = female
Age	Respondent's age
Age squared	Age squared
University degree	Binary variable: 1 = obtained university degree and 0 = otherwise
Marriage	Binary variable: 1 = married and 0 = otherwise
Divorce	Binary variable: 1 = divorced or separated and 0 = otherwise
Household members	Continuous variable: number of people currently living in household
Children	Binary variable: 1 = have child/children and 0 = otherwise
Unemployed	Binary variable: 1 = respondent is unemployed and 0 = otherwise
Household income	Continuous variable: annual earned income before taxes and with bonuses of the entire household in 2009 (unit: JPY)
Log of household income	Log (household income)
Household assets	Continuous variable: balance of financial assets (savings, stock, insurance, etc.) of the entire household (unit: JPY)
Log of household assets	Log (household assets)
Regular exercise	Binary variable: 1 = regular exercise (exercise once a week or more) and 0 = otherwise
Current drinker	Binary variable: 1 = current drinker (drink sometimes–five cans of beer daily) and 0 = otherwise
Frequent gambler	Binary variable: 1 = frequent gambler (gamble once a week or more) and 0 = otherwise
Myopic view of the future	Binary variable: 1 = agree and completely agree with the statement “Since the future is uncertain, it is a waste to think about it” and 0 = otherwise
Level of risk preference	Continuous variable: percentage score from the question “Usually, when you go outdoors, how high does the probability of rain have to be before you take an umbrella?”
Current level of happiness	Continuous variable: percentage score from the question “Overall, how happy would you say you are currently?”
Anxiety about health	Binary variable: 1 = agree and completely agree with the statement “I have anxieties about my health” and 0 = otherwise

### Descriptive Statistics

The descriptive statistics in Table 2 show that 24.26% of respondents were current smokers. On average, respondents' financial literacy scores were 0.59 and 15.33% of the sample received financial education at school. For the demographic variables, about 49.24% of the sample were men and the average age was 49.79 years. Approximately 27.06% of the sample hold a university degree, 82.25% of the sample were currently married, and 3.45% were divorced. The respondents had four household members on average, and about 84.43% of the sample had children. Only 2.40% of the sample were currently unemployed. Respondents had an annual household income of ~6.49 million JPY on average and 13.10 million JPY in household assets in 2009. For risky behaviors, 37.13% of the participants exercised regularly, whilst 54.16% were current drinkers and 9.42% were frequent gamblers. Overall, 14.54% of the respondents had a myopic view of the future and risk preferences of 49.08%; in other words, they were risk neutral. Respondents rated their current level of happiness at 64.72%, and 41.99% of the sample were anxious about their health.

**Table 2:** Descriptive statistics.

**Variables**	**Mean**	**Standard deviation (SD)**	**Min**	**Max**
Smoking behavior	0.2426	0.4287	0	1
Financial literacy	0.5914	0.3436	0	1
Financial education	0.1533	0.3603	0	1
Male	0.4924	0.5000	0	1
Age	49.79	12.61	20	76
Age squared	2637.63	1239.67	400	5,776
University degree	0.2706	0.4444	0	1
Marriage	0.8225	0.3822	0	1
Divorce	0.0345	0.1826	0	1
Household members	3.52	1.44	1	10
Children	0.8443	0.3626	0	1
Unemployed	0.0240	0.1531	0	1
Household income	6,486,239	3,777,635	1,000,000	20,000,000
Log of household income	15.51	0.61	13.82	16.81
Household assets	13,100,000	17,600,000	2,500,000	100,000,000
Log of household assets	15.81	1.01	14.73	18.42
Regular exercise	0.3713	0.4832	0	1
Current drinker	0.5416	0.4983	0	1
Frequent gambler	0.0942	0.2921	0	1
Myopic view of the future	0.1454	0.3526	0	1
Level of risk preference	0.4908	0.1891	0	1
Current level of happiness	0.6472	0.1822	0	1
Anxiety about health	0.4199	0.4936	0	1
Observations	3,706			

Tables 3–5 present the distribution of smoking behavior classified by age group, demographic characteristics, and risky behaviors, respectively. Our sample contained 899 current smokers; that is, 24.26% of the total sample smoke between sometimes to more than two packs of cigarettes daily, whilst the remaining 2,807 respondents were non-smokers. The results in Table 3 indicate significant differences in smoking behavior among age groups. The proportion of current smokers in the oldest age group (age 61 and older) was 17.27%, which is less than other age groups that the proportions of current smokers are more than 22%. In Table 4, we see significant differences in smoking behavior between genders. Approximately 37.15% of male respondents were current smokers compared to 11.75% of female respondents. However, the differences in smoking behavior by education level and employment status are insignificant. The results in Table 5 for risky behaviors show that about 19.11% of respondents who exercise regularly are current smokers, which is less than the sample of current smokers who do not exercise regularly (27.30%). In addition, we see considerable differences in smoking behavior between current drinkers and non-drinkers and between frequent gamblers and non-gamblers. Specifically, 30.44% of current drinkers and 41.55% of frequent gamblers were current smokers.

**Table 3:** Distribution of smoking behavior by age group.

**Smoking behavior**	**Age**					**Total**
	**≤30**	**31–40**	**41–50**	**51–60**	**≥61**	
Non-smokers	212	496	679	716	704	2,807
	77.94%	71.78%	73.09%	74.35%	82.73%	75.74%
Current smokers	60	195	250	247	147	899
	22.06%	28.22%	26.91%	25.65%	17.27%	24.26%
Total	272	691	929	963	851	3,706
	100%	100%	100%	100%	100%	100%
Mean difference	*F* = 8.51^***^					

*** p < 0.01,

** p < 0.05,

* *p < 0.10*.

**Table 4:** Distribution of smoking behavior by demographic characteristic.

**Smoking behavior**	**Gender**		**Education**		**Unemployed**		**Total**
	**Female**	**Male**	**Lower than university degree**	**University degree and higher**	**No**	**Yes**	
Non-smokers	1,660	1,147	2,035	772	2,743	64	2,807
	88.25%	62.85%	75.29%	76.97%	75.84%	71.91%	75.74%
Current smokers	221	678	668	231	874	25	899
	11.75%	37.15%	24.71%	23.03%	24.16%	28.09%	24.26%
Total	1,881	1,825	2,703	1,003	3,617	89	3,706
	100%	100%	100%	100%	100%	100%	100%
Mean difference	*t* = −18.8786^***^		*t* = 1.0614		*t* = −0.8535		

*** p < 0.01,

** p < 0.05,

* p < 0.10.

**Table 5:** Distribution of smoking behavior by risky behaviors.

**Smoking behavior**	**Regular exercise**		**Current drinkers**		**Frequent gamblers**		**Total**
	**No**	**Yes**	**No**	**Yes**	**No**	**Yes**	
Non-smokers	1,694	1,113	1,411	1,396	2,603	204	2,807
	72.70%	80.89%	83.05%	69.56%	77.54%	58.45%	75.74%
Current smokers	636	263	288	611	754	145	899
	27.30%	19.11%	16.95%	30.44%	22.46%	41.55%	24.26%
Total	2,330	1,376	1,699	2,007	3,357	349	3,706
	100%	100%	100%	100%	100%	100%	100%
Mean difference	*t* = 5.6374^***^		*t* = −9.6649^***^		*t* = −7.9829^***^		

*** p < 0.01,

** p < 0.05,

* p < 0.10.

## Methodology

To investigate how financial literacy and financial education are related to smoking behavior, we first separately estimate the effects of financial literacy and financial education in Equations (1, 2), respectively. We then include both financial literacy and financial education to see the combined effect of the variables in Equation (3).

(1) $$Y_{i}=f(FL_{i},X_{i},\varepsilon_{i})$$

(2) $$Y_{i}=f(FE_{i},X_{i},\varepsilon_{i})$$

(3) $$Y_{i}=f(FL_{i},FE_{i},X_{i},\varepsilon_{i})$$

where Y_i_ is the smoking behavior of the *i*th respondent (current smokers or non-smokers), FL represents the score on the financial questions measuring financial literacy, FE represents financial education received at school, X is a vector of individual characteristics, and ε is the error term. Because the dependent variable is a binary choice, we employ a probit regression to estimate all equations.

As there is a potential for multicollinearity between the explanatory variables in the models (i.e., individuals with a high level of education could have high financial knowledge, or individuals with high net worth may have more financial knowledge because of experience with asset management), we conducted correlation and multicollinearity tests in all models (available upon request). The results show that multicollinearity between the variables is not significant, suggesting that the independent effects of explanatory variables on smoking behavior are not biased. The correlation matrix shows a weak relationship between the explanatory variables (lower than 0.70). In addition, the variance inflation factor (VIF) tests of the explanatory variables are below 10, indicating that multicollinearity is not significant in all models.

The full model specifications are

*Smoking behavior*_*i*_ (1 = *current smokers and* 0 = *non*−*smokers*) = β_0_ + β_1_*financial literacy*_*i*_ + β_2_*male*_*i*_ + β_3_*age*_*i*_ + β_4_*age squared*_*i*_ + β_5_*university degree*_*i*_ + β_6_*marriage*_*i*_ + β_7_*divorce*_*i*_ + _β_8_*household members*_*i*_ + β9_*children*_*i*_ + _β_10_*unemployed*_*i*_ + β11_*log of household income*_*i*_ + β_12_ *logofhousehold assets*_*i*_ + β_13_*regular exercise*_*i*_ + β_14_*current drinkers*_*i*_ + β_15_*frequent gamblers*_*i*_ + β_16_*myopic view of the future*_*i*_ + β_17_*level of risk preference*_*i*_ + β_18_*current level of happiness*_*i*_ + β_19_*anxiety about health*_*i*_ + ε_*i*_ (1a)

*Smoking behavior*_*i*_ (1 = *current smokers and* 0 = *non*−*smokers*) = β_0_ + β_1_*financial education*_*i*_ + β_2_*male*_*i*_ + β_3_*age*_*i*_ + β_4_*age squared*_*i*_ + β_5_*university degree*_*i*_ + β_6_*marriage*_*i*_ + β_7_*divorce*_*i*_ + _β_8_*household members*_*i*_ + β9_*children*_*i*_ + _β_10_*unemployed*_*i*_ + β11_*log of household income*_*i*_ + β_12_ *logofhousehold assets*_*i*_ + β_13_*regular exercise*_*i*_ + β_14_*current drinkers*_*i*_ + β_15_*frequent gamblers*_*i*_ + β_16_*myopic view of the future*_*i*_ + β_17_*level of risk preference*_*i*_ + β_18_*current level of happiness*_*i*_ + β_19_*anxiety about health*_*i*_ + ε_**i**_ (2a)

*Smoking behavior*_*i*_ (1 = *current smokers and* 0 = *non*−*smokers*) = β_0_ + β_1_*financial literacy*_*i*_ + β_2_*financial education*_*i*_ + β_3_*male*_*i*_ + β_4_*age*_*i*_ + β_5_*age squared*_*i*_ + β_6_*university degree*_*i*_ + β_7_*marriage*_*i*_ + β_8_*divorce*_*i*_ + _β_9_*household members*_*i*_ + β10_*children*_*i*_ + _β_11_*unemployed*_*i*_ + β12_*log of household income*_*i*_ + β_13_ *logofhousehold assets*_*i*_ + β_14_*regular exercise*_*i*_ + β_15_*current drinkers*_*i*_ + β_16_*frequent gamblers*_*i*_ + β_17_*myopic view of the future*_*i*_ + β_18_*level of risk preference*_*i*_ + β_19_*current level of happiness*_*i*_ + β_20_*anxiety about health*_*i*_ + ε_**i**_ (3a)

## Results

We present the results of the probit regression to estimate Equations (1)-(3) in Tables 6–8, respectively. Each table presents the results of four different specifications of the explanatory variables. The first specification (Models 1.1, 2.1, and 3.1) included controls for only the demographic variables. In the second specification (Models 1.2, 2.2, and 3.2), we added risky behaviors including exercise, drinking alcohol, and gambling. The third specification (Models 1.3, 2.3, and 3.3) included respondents' myopic views of the future and risk preferences. Finally, the fourth specification (Models 1.4, 2.4, and 3.4) included respondents' self-rated level of happiness and anxiety about health.

**Table 6:** Probit model regression results, financial literacy as the main explanatory variable.

**Variables**	**Dependent variable: smoking behavior**			
	**Model 1.1**	**Model 1.2**	**Model 1.3**	**Model 1.4**
Financial literacy	−0.228^***^	−0.209^***^	−0.181^**^	−0.165^**^
	(0.0759)	(0.0767)	(0.0772)	(0.0770)
Male	1.018^***^	0.940^***^	0.928^***^	0.909^***^
	(0.0523)	(0.0558)	(0.0559)	(0.0562)
Age	0.0765^***^	0.0653^***^	0.0673^***^	0.0621^***^
	(0.0153)	(0.0152)	(0.0153)	(0.0154)
Age squared	−0.000892^***^	−0.000769^***^	−0.000787^***^	−0.000750^***^
	(0.000153)	(0.000153)	(0.000154)	(0.000155)
University degree	−0.260^***^	−0.250^***^	−0.231^***^	−0.214^***^
	(0.0589)	(0.0593)	(0.0596)	(0.0599)
Marriage	−0.0610	−0.0466	−0.0428	0.0164
	(0.105)	(0.105)	(0.105)	(0.108)
Divorce	0.441^***^	0.449^***^	0.438^***^	0.453^***^
	(0.154)	(0.153)	(0.154)	(0.156)
Household members	−0.0154	−0.0201	−0.0212	−0.0282
	(0.0199)	(0.0200)	(0.0201)	(0.0202)
Children	0.0496	0.0452	0.0532	0.0707
	(0.105)	(0.106)	(0.106)	(0.107)
Unemployed	−0.0720	−0.0216	−0.0185	−0.0863
	(0.160)	(0.158)	(0.158)	(0.161)
Log of household income	−0.109^**^	−0.120^**^	−0.118^**^	−0.0829^*^
	(0.0472)	(0.0476)	(0.0477)	(0.0485)
Log of household assets	−0.0794^***^	−0.0590^**^	−0.0551^*^	−0.0421
	(0.0290)	(0.0293)	(0.0294)	(0.0296)
Regular exercise		−0.281^***^	−0.275^***^	−0.246^***^
		(0.0527)	(0.0528)	(0.0531)
Current drinker		0.231^***^	0.230^***^	0.235^***^
		(0.0523)	(0.0524)	(0.0525)
Frequent gambler		0.282^***^	0.265^***^	0.256^***^
		(0.0769)	(0.0769)	(0.0774)
Myopic view of the future			0.172^**^	0.156^**^
			(0.0674)	(0.0676)
Level of risk preference			0.424^***^	0.435^***^
			(0.129)	(0.130)
Current level of happiness				−0.684^***^
				(0.142)
Anxiety about health				0.00801
				(0.0504)
Constant	0.446	0.502	0.0839	−0.121
	(0.741)	(0.746)	(0.753)	(0.755)
Observations	3,706	3,706	3,706	3,706
Log likelihood	−1803	−1773	−1764	−1753
Chi2 statistics	466.7	505.4	516	524.4
*p*-value	0	0	0	0

Robust standard errors in parentheses,

*** p < 0.01,

** p < 0.05,

* *p < 0.1*.

**Table 7:** Probit model regression results, financial education as the main explanatory variable.

**Variables**	**Dependent variable: smoking behavior**			
	**Model 2.1**	**Model 2.2**	**Model 2.3**	**Model 2.4**
Financial education	−0.139^**^	−0.114	−0.118^*^	−0.127^*^
	(0.0693)	(0.0698)	(0.0700)	(0.0701)
Male	0.995^***^	0.920^***^	0.910^***^	0.893^***^
	(0.0518)	(0.0553)	(0.0553)	(0.0557)
Age	0.0735^***^	0.0623^***^	0.0652^***^	0.0602^***^
	(0.0152)	(0.0151)	(0.0152)	(0.0153)
Age squared	−0.000862^***^	−0.000740^***^	−0.000765^***^	−0.000730^***^
	(0.000152)	(0.000152)	(0.000153)	(0.000154)
University degree	−0.291^***^	−0.279^***^	−0.254^***^	−0.234^***^
	(0.0584)	(0.0588)	(0.0591)	(0.0594)
Marriage	−0.0645	−0.0499	−0.0451	0.0163
	(0.105)	(0.106)	(0.106)	(0.108)
Divorce	0.427^***^	0.437^***^	0.427^***^	0.444^***^
	(0.154)	(0.153)	(0.154)	(0.155)
Household members	−0.00888	−0.0142	−0.0160	−0.0235
	(0.0199)	(0.0200)	(0.0201)	(0.0202)
Children	0.0629	0.0570	0.0639	0.0811
	(0.105)	(0.106)	(0.106)	(0.107)
Unemployed	−0.0538	−0.00506	−0.00241	−0.0731
	(0.160)	(0.158)	(0.158)	(0.160)
Log of household income	−0.131^***^	−0.140^***^	−0.134^***^	−0.0971^**^
	(0.0467)	(0.0472)	(0.0473)	(0.0481)
Log of household assets	−0.0894^***^	−0.0679^**^	−0.0625^**^	−0.0485^*^
	(0.0288)	(0.0290)	(0.0291)	(0.0293)
Regular exercise		−0.284^***^	−0.278^***^	−0.247^***^
		(0.0527)	(0.0528)	(0.0531)
Current drinker		0.230^***^	0.228^***^	0.233^***^
		(0.0523)	(0.0524)	(0.0525)
Frequent gambler		0.278^***^	0.260^***^	0.249^***^
		(0.0769)	(0.0768)	(0.0774)
Myopic view of the future			0.179^***^	0.161^**^
			(0.0674)	(0.0677)
Level of risk preference			0.451^***^	0.460^***^
			(0.129)	(0.130)
Current level of happiness				−0.702^***^
				(0.142)
Anxiety about health				0.0123
				(0.0504)
Constant	0.879	0.895	0.395	0.152
	(0.723)	(0.728)	(0.737)	(0.739)
Observations	3,706	3,706	3,706	3,706
Log likelihood	−1806	−1776	−1766	−1753
Chi2 statistics	463.4	506.3	519.1	529.7
*p*-value	0	0	0	0

Robust standard errors in parentheses,

*** p < 0.01,

** p < 0.05,

* *p < 0.1*.

**Table 8:** Probit model regression results, financial literacy, and financial education as the main explanatory variables.

**Variables**	**Dependent variable: smoking behavior**			
	**Model 3.1**	**Model 3.2**	**Model 3.3**	**Model 3.4**
Financial literacy	−0.227^***^	−0.208^***^	−0.180^**^	−0.164^**^
	(0.0760)	(0.0768)	(0.0772)	(0.0771)
Financial education	−0.138^**^	−0.113	−0.116^*^	−0.125^*^
	(0.0692)	(0.0698)	(0.0699)	(0.0700)
Male	1.018^***^	0.941^***^	0.928^***^	0.909^***^
	(0.0524)	(0.0558)	(0.0559)	(0.0562)
Age	0.0774^***^	0.0661^***^	0.0683^***^	0.0631^***^
	(0.0153)	(0.0153)	(0.0153)	(0.0154)
Age squared	−0.000896^***^	−0.000773^***^	−0.000792^***^	−0.000755^***^
	(0.000153)	(0.000153)	(0.000154)	(0.000155)
University degree	−0.259^***^	−0.250^***^	−0.231^***^	−0.214^***^
	(0.0590)	(0.0593)	(0.0597)	(0.0600)
Marriage	−0.0570	−0.0436	−0.0395	0.0201
	(0.105)	(0.105)	(0.105)	(0.108)
Divorce	0.439^***^	0.448^***^	0.437^***^	0.452^***^
	(0.154)	(0.153)	(0.154)	(0.155)
Household members	−0.0140	−0.0188	−0.0199	−0.0269
	(0.0199)	(0.0201)	(0.0201)	(0.0202)
Children	0.0503	0.0460	0.0538	0.0716
	(0.105)	(0.106)	(0.106)	(0.107)
Unemployed	−0.0668	−0.0174	−0.0134	−0.0816
	(0.159)	(0.157)	(0.158)	(0.161)
Log of household income	−0.111^**^	−0.121^**^	−0.119^**^	−0.0837^*^
	(0.0472)	(0.0476)	(0.0476)	(0.0484)
Log of household assets	−0.0791^***^	−0.0589^**^	−0.0549^*^	−0.0418
	(0.0291)	(0.0294)	(0.0294)	(0.0296)
Regular exercise		−0.279^***^	−0.273^***^	−0.244^***^
		(0.0527)	(0.0528)	(0.0532)
Current drinker		0.229^***^	0.228^***^	0.232^***^
		(0.0524)	(0.0525)	(0.0526)
Frequent gambler		0.277^***^	0.259^***^	0.250^***^
		(0.0769)	(0.0769)	(0.0774)
Myopic view of the future			0.169^**^	0.152^**^
			(0.0676)	(0.0678)
Level of risk preference			0.433^***^	0.444^***^
			(0.129)	(0.130)
Current level of happiness				−0.689^***^
				(0.142)
Anxiety about health				0.0105
				(0.0505)
Constant	0.438	0.492	0.0665	−0.142
	(0.741)	(0.746)	(0.753)	(0.755)
Observations	3,706	3,706	3,706	3,706
Log likelihood	−1801	−1772	−1763	−1751
Chi2 statistics	471.4	510.4	522.3	531.9
*p*-value	0	0	0	0

Robust standard errors in parentheses,

*** p < 0.01,

** p < 0.05,

* *p < 0.1*.

The results in Table 6 show that financial literacy has a negative and strongly significant impact on smoking behavior across the models. In Table 7, we see that financial education also has a negative and significant impact on smoking behavior in all models except Model 2.2. However, the significance levels of the financial education variables (at 5 and 10%) are less than those of the financial literacy variables (at % and 5%). Since both financial literacy and financial education have a significant impact on smoking behavior, we regress financial literacy on financial education and the other control variables to explore the relationship between these two variables. We find that financial literacy and financial education are not correlated (results not reported here, but available upon request), consistent with the finding of Sekita (71). Therefore, we should use both financial literacy and financial education as explanatory variables in the same equation to explain smoking behavior, as shown in our final model in Table 8.

The results in Table 8 show that, overall, there are no differences in the significance of the estimated parameters compared to the results in Tables 6, 7. The coefficients of our variables of interest, financial literacy, and financial education are negative and statistically significant in all models except financial education in Model 3.2. In other words, respondents with a high level of financial literacy and those who received financial education were less likely to be current smokers at present.

For the demographic and other control variables, most of the signs and significance levels of the coefficients are consistent across models and specifications. Male, age, and divorce have a positive impact on being a current smoker, and the coefficients are strongly significant at the 1% level. In contrast, age squared, university degree, log of household income, and log of household assets have a negative and significant impact on being a current smoker. However, marriage, household members, children, and employment status have an insignificant impact. In terms of risky behaviors, regular exercise has a negative impact, whilst being a current drinker and frequent gambler have a positive impact on current smoking status, statistically significant at the 1% level. Furthermore, a myopic view of the future and a high level of risk preference are associated with being current smokers. Conversely, respondents with self-rated high levels of happiness are less likely to be current smokers. However, respondents' anxiety regarding health showed an insignificant impact on smoking behavior.

## Robustness Check

To check robustness of our results, we used an alternative classification of smoking behavior. Rather than classifying respondents as non-smokers and smokers, we classified them as non-smokers, occasional or intermittent smokers, and regular smokers. The alternative classification allowed us to check rationality in smoking behavior elaborately. Non-smokers included respondents who answered “do not smoke at all” and “I used to smoke but had quit.” Occasional smokers included respondents who answered “hardly smoke” and “smoke sometimes.” Regular smokers included respondents who answered “about 10 cigarettes a day,” “about a pack a day,” and “more than two packs a day.” Similar to our original models, we hypothesize that respondents who are financially literate or received more financial education are less likely to be regular smokers.

We used multinomial probit model (mprobit) to investigate how financial literacy and financial education are related to smoking behavior. The results of mprobit regressions are presented in Tables 9–11. Each table presents the results of four different specifications of the explanatory variables where the base model is non-smokers' category. The results in Table 9 show that financial literacy has a negative and significant impact on smoking behavior in all models except Model 4.7 in case of occasional smokers. The results in Table 10 indicate that financial education also has a negative and significant impact on smoking behavior in regular smokers' category. We included both financial literacy and financial education as explanatory variables in our final model. The results in Table 11 show that, overall, there are no differences in the significance of the estimated parameters compared with the results in Tables 9, 10. The coefficients of our variables of interest, financial literacy and financial education, are negative and statistically significant across models in case of the regular smokers. In other words, compared with non-smokers, respondents with a high level of financial literacy and those who received financial education are less likely to be regular smokers. These results are similar to the probit estimation when smoking behavior is classified into the non-smokers and smokers categories.

**Table 9:** Multinomial probit model regression results, financial literacy as the main explanatory variable.

**Variables**	**Occasional smoker**	**Regular smoker**	**Occasional smoker**	**Regular smoker**	**Occasional smoker**	**Regular smoker**	**Occasional smoker**	**Regular smoker**
	**Model 4.1**	**Model 4.2**	**Model 4.3**	**Model 4.4**	**Model 4.5**	**Model 4.6**	**Model 4.7**	**Model 4.8**
Financial literacy	−0.269^*^	−0.316^***^	−0.257^*^	−0.291^***^	−0.243^*^	−0.247^**^	−0.234	−0.224^**^
	(0.143)	(0.109)	(0.142)	(0.110)	(0.143)	(0.110)	(0.142)	(0.110)
Male	0.643^***^	1.468^***^	0.620^***^	1.355^***^	0.612^***^	1.336^***^	0.594^***^	1.309^***^
	(0.0979)	(0.0750)	(0.106)	(0.0797)	(0.106)	(0.0798)	(0.107)	(0.0802)
Age	0.0139	0.116^***^	0.0103	0.0999^***^	0.0110	0.104^***^	0.00670	0.0962^***^
	(0.0266)	(0.0222)	(0.0267)	(0.0222)	(0.0267)	(0.0222)	(0.0269)	(0.0223)
Age squared	−0.000259	−0.00133^***^	−0.000218	−0.00116^***^	−0.000230	−0.00119^***^	−0.000198	−0.00114^***^
	(0.000271)	(0.000224)	(0.000272)	(0.000224)	(0.000271)	(0.000224)	(0.000272)	(0.000225)
University degree	−0.195^*^	−0.396^***^	−0.208^*^	−0.382^***^	−0.199^*^	−0.351^***^	−0.185	−0.327^***^
	(0.115)	(0.0842)	(0.115)	(0.0847)	(0.115)	(0.0852)	(0.115)	(0.0857)
Marriage	0.0299	−0.118	0.0228	−0.0954	0.0257	−0.0877	0.0736	−0.00118
	(0.173)	(0.151)	(0.176)	(0.150)	(0.175)	(0.152)	(0.177)	(0.154)
Divorce	0.482^*^	0.598^***^	0.465^*^	0.612^***^	0.455	0.597^***^	0.472^*^	0.621^***^
	(0.280)	(0.220)	(0.280)	(0.219)	(0.280)	(0.220)	(0.280)	(0.221)
Household member	0.0408	−0.0131	0.0365	−0.0195	0.0360	−0.0219	0.0309	−0.0320
	(0.0385)	(0.0283)	(0.0384)	(0.0286)	(0.0384)	(0.0286)	(0.0385)	(0.0287)
Children	−0.275	0.0652	−0.271	0.0562	−0.262	0.0669	−0.251	0.0924
	(0.175)	(0.151)	(0.176)	(0.151)	(0.176)	(0.152)	(0.177)	(0.153)
Unemployed	−0.315	−0.0875	−0.303	−0.0120	−0.302	−0.00560	−0.352	−0.101
	(0.331)	(0.230)	(0.332)	(0.228)	(0.333)	(0.229)	(0.339)	(0.232)
Log of household income	−0.117	−0.140^**^	−0.116	−0.156^**^	−0.115	−0.152^**^	−0.0861	−0.102
	(0.0928)	(0.0670)	(0.0931)	(0.0675)	(0.0932)	(0.0676)	(0.0939)	(0.0691)
Log of household assets	−0.0541	−0.115^***^	−0.0456	−0.0864^**^	−0.0439	−0.0792^*^	−0.0346	−0.0609
	(0.0538)	(0.0413)	(0.0543)	(0.0417)	(0.0546)	(0.0418)	(0.0549)	(0.0420)
Regular exercise			−0.145	−0.386^***^	−0.140	−0.376^***^	−0.120	−0.335^***^
			(0.0973)	(0.0754)	(0.0975)	(0.0756)	(0.0980)	(0.0761)
Current drinker			0.162	0.329^***^	0.166^*^	0.328^***^	0.169^*^	0.334^***^
			(0.101)	(0.0748)	(0.100)	(0.0750)	(0.101)	(0.0752)
Frequent gambler			−0.193	0.396^***^	−0.197	0.368^***^	−0.203	0.355^***^
			(0.175)	(0.109)	(0.175)	(0.110)	(0.175)	(0.110)
Myopic view of the future					0.211^*^	0.254^***^	0.200	0.233^**^
					(0.126)	(0.0967)	(0.126)	(0.0971)
Level of risk preference					0.0796	0.712^***^	0.0883	0.726^***^
					(0.248)	(0.185)	(0.248)	(0.186)
Current level of happiness							−0.548^**^	−0.980^***^
							(0.271)	(0.203)
Anxiety about health							−0.00979	−0.00885
							(0.0955)	(0.0721)
Constant	0.692	0.196	0.622	0.283	0.485	−0.423	0.330	−0.696
	(1.373)	(1.057)	(1.375)	(1.065)	(1.387)	(1.076)	(1.384)	(1.080)
Observations	3,706	3,706	3,706	3,706	3,706	3,706	3,706	3,706
Log likelihood	−2433	−2433	−2402	−2402	−2390	−2390	−2378	−2378
Chi2 statistics	487.4	487.4	535.1	535.1	550.6	550.6	557.3	557.3
p-value	0	0	0	0	0	0	0	0

Robust standard errors in parentheses,

*** p < 0.01,

** p < 0.05,

* *p < 0.1*.

**Table 10:** Multinomial probit model regression results, financial education as the main explanatory variable.

**Variables**	**Occasional smoker**	**Regular smoker**	**Occasional smoker**	**Regular smoker**	**Occasional smoker**	**Regular smoker**	**Occasional smoker**	**Regular smoker**
	**Model 5.1**	**Model 5.2**	**Model 5.3**	**Model 5.4**	**Model 5.5**	**Model 5.6**	**Model 5.7**	**Model 5.8**
Financial education	0.0365	−0.215^**^	0.0392	−0.178^*^	0.0464	−0.186^*^	0.0426	−0.198^**^
	(0.128)	(0.0996)	(0.128)	(0.100)	(0.128)	(0.101)	(0.128)	(0.101)
Male	0.619^***^	1.436^***^	0.597^***^	1.328^***^	0.590^***^	1.313^***^	0.573^***^	1.288^***^
	(0.0967)	(0.0744)	(0.105)	(0.0791)	(0.105)	(0.0792)	(0.106)	(0.0797)
Age	0.00902	0.112^***^	0.00534	0.0958^***^	0.00639	0.101^***^	0.00224	0.0936^***^
	(0.0265)	(0.0221)	(0.0265)	(0.0221)	(0.0265)	(0.0221)	(0.0267)	(0.0222)
Age squared	−0.000218	−0.00129^***^	−0.000175	−0.00112^***^	−0.000191	−0.00116^***^	−0.000160	−0.00111^***^
	(0.000271)	(0.000222)	(0.000271)	(0.000222)	(0.000270)	(0.000223)	(0.000271)	(0.000224)
University degree	−0.232^**^	−0.439^***^	−0.242^**^	−0.421^***^	−0.230^**^	−0.382^***^	−0.213^*^	−0.354^***^
	(0.112)	(0.0836)	(0.113)	(0.0840)	(0.112)	(0.0846)	(0.113)	(0.0850)
Marriage	0.0211	−0.121	0.0149	−0.0977	0.0187	−0.0885	0.0681	0.00139
	(0.175)	(0.152)	(0.177)	(0.151)	(0.176)	(0.152)	(0.178)	(0.155)
Divorce	0.469^*^	0.580^***^	0.454	0.598^***^	0.444	0.585^***^	0.461^*^	0.611^***^
	(0.280)	(0.219)	(0.280)	(0.219)	(0.280)	(0.220)	(0.280)	(0.221)
Household member	0.0464	−0.00413	0.0416	−0.0114	0.0404	−0.0148	0.0351	−0.0255
	(0.0383)	(0.0283)	(0.0382)	(0.0286)	(0.0383)	(0.0286)	(0.0384)	(0.0287)
Children	−0.260	0.0835	−0.257	0.0725	−0.249	0.0812	−0.237	0.106
	(0.175)	(0.151)	(0.176)	(0.151)	(0.177)	(0.152)	(0.177)	(0.153)
Unemployed	−0.312	−0.0624	−0.299	0.0108	−0.299	0.0167	−0.350	−0.0826
	(0.328)	(0.230)	(0.329)	(0.227)	(0.330)	(0.227)	(0.336)	(0.231)
Log of household income	−0.140	−0.170^**^	−0.139	−0.182^***^	−0.136	−0.174^***^	−0.105	−0.121^*^
	(0.0921)	(0.0662)	(0.0924)	(0.0669)	(0.0925)	(0.0670)	(0.0931)	(0.0684)
Log of household assets	−0.0660	−0.129^***^	−0.0564	−0.0991^**^	−0.0540	−0.0895^**^	−0.0440	−0.0697^*^
	(0.0536)	(0.0410)	(0.0540)	(0.0413)	(0.0543)	(0.0414)	(0.0546)	(0.0417)
Regular exercise			−0.156	−0.390^***^	−0.150	−0.379^***^	−0.128	−0.336^***^
			(0.0975)	(0.0754)	(0.0977)	(0.0755)	(0.0981)	(0.0761)
Current drinker			0.162	0.326^***^	0.166^*^	0.324^***^	0.169^*^	0.330^***^
			(0.101)	(0.0747)	(0.100)	(0.0749)	(0.101)	(0.0752)
Frequent gambler			−0.189	0.391^***^	−0.193	0.360^***^	−0.200	0.346^***^
			(0.175)	(0.109)	(0.175)	(0.109)	(0.175)	(0.110)
Myopic view of the future					0.223^*^	0.262^***^	0.211^*^	0.239^**^
					(0.125)	(0.0968)	(0.125)	(0.0973)
Level of risk preference					0.102	0.750^***^	0.111	0.764^***^
					(0.249)	(0.184)	(0.249)	(0.186)
Current level of happiness							−0.559^**^	−1.005^***^
							(0.271)	(0.203)
Anxiety about health							−0.00679	−0.00260
							(0.0953)	(0.0721)
Constant	1.212	0.799	1.120	0.830	0.932	−0.00556	0.754	−0.332
	(1.353)	(1.031)	(1.354)	(1.040)	(1.366)	(1.054)	(1.365)	(1.058)
Observations	3,706	3,706	3,706	3,706	3,706	3,706	3,706	3,706
Log likelihood	−2435	−2435	−2404	−2404	−2391	−2391	−2379	−2379
Chi2 statistics	485.3	485.3	535.9	535.9	554.2	554.2	563.6	563.6
*p*-value	0	0	0	0	0	0	0	0

Robust standard errors in parentheses,

*** p < 0.01,

** p < 0.05,

* *p < 0.1*.

**Table 11:** Multinomial probit model regression results, financial literacy, and financial education as the main explanatory variables.

**Variables**	**Occasional smoker**	**Regular smoker**	**Occasional smoker**	**Regular smoker**	**Occasional smoker**	**Regular smoker**	**Occasional smoker**	**Regular smoker**
	**Model 6.1**	**Model 6.2**	**Model 6.3**	**Model 6.4**	**Model 6.5**	**Model 6.6**	**Model 6.7**	**Model 6.8**
Financial literacy	−0.268^*^	−0.314^***^	−0.256^*^	−0.290^***^	−0.242^*^	−0.245^**^	−0.233	−0.222^**^
	(0.143)	(0.109)	(0.142)	(0.110)	(0.143)	(0.111)	(0.142)	(0.110)
Financial education	0.0386	−0.213^**^	0.0408	−0.176^*^	0.0480	−0.184^*^	0.0442	−0.196^*^
	(0.128)	(0.0995)	(0.128)	(0.100)	(0.128)	(0.101)	(0.128)	(0.100)
Male	0.644^***^	1.468^***^	0.621^***^	1.357^***^	0.613^***^	1.337^***^	0.595^***^	1.310^***^
	(0.0979)	(0.0750)	(0.106)	(0.0796)	(0.106)	(0.0797)	(0.107)	(0.0802)
Age	0.0138	0.118^***^	0.0101	0.101^***^	0.0107	0.105^***^	0.00644	0.0977^***^
	(0.0266)	(0.0223)	(0.0267)	(0.0222)	(0.0267)	(0.0222)	(0.0269)	(0.0224)
Age squared	−0.000260	−0.00134^***^	−0.000217	−0.00116^***^	−0.000229	−0.00120^***^	−0.000197	−0.00114^***^
	(0.000271)	(0.000224)	(0.000272)	(0.000224)	(0.000272)	(0.000224)	(0.000272)	(0.000225)
University degree	−0.197^*^	−0.395^***^	−0.208^*^	−0.381^***^	−0.200^*^	−0.350^***^	−0.185	−0.326^***^
	(0.115)	(0.0844)	(0.115)	(0.0848)	(0.115)	(0.0853)	(0.115)	(0.0858)
Marriage	0.0309	−0.110	0.0237	−0.0892	0.0268	−0.0810	0.0746	0.00650
	(0.174)	(0.151)	(0.176)	(0.150)	(0.175)	(0.152)	(0.177)	(0.154)
Divorce	0.484^*^	0.597^***^	0.467^*^	0.612^***^	0.457	0.597^***^	0.474^*^	0.622^***^
	(0.280)	(0.220)	(0.280)	(0.219)	(0.280)	(0.220)	(0.280)	(0.221)
Household member	0.0401	−0.0111	0.0359	−0.0177	0.0352	−0.0200	0.0302	−0.0301
	(0.0384)	(0.0284)	(0.0383)	(0.0287)	(0.0384)	(0.0287)	(0.0385)	(0.0288)
Children	−0.276	0.0655	−0.272	0.0566	−0.263	0.0668	−0.252	0.0927
	(0.175)	(0.151)	(0.176)	(0.151)	(0.177)	(0.151)	(0.178)	(0.153)
Unemployed	−0.322	−0.0793	−0.308	−0.00519	−0.308	0.00268	−0.357	−0.0933
	(0.330)	(0.229)	(0.331)	(0.227)	(0.333)	(0.228)	(0.338)	(0.231)
Log of household income	−0.116	−0.142^**^	−0.116	−0.157^**^	−0.114	−0.153^**^	−0.0856	−0.103
	(0.0924)	(0.0670)	(0.0927)	(0.0675)	(0.0928)	(0.0676)	(0.0935)	(0.0691)
Log of household assets	−0.0540	−0.114^***^	−0.0454	−0.0863^**^	−0.0437	−0.0790^*^	−0.0343	−0.0605
	(0.0537)	(0.0414)	(0.0542)	(0.0418)	(0.0545)	(0.0419)	(0.0547)	(0.0421)
Regular exercise			−0.146	−0.383^***^	−0.142	−0.373^***^	−0.121	−0.331^***^
			(0.0972)	(0.0755)	(0.0975)	(0.0756)	(0.0979)	(0.0761)
Current drinker			0.163	0.325^***^	0.166^*^	0.324^***^	0.170^*^	0.330^***^
			(0.101)	(0.0749)	(0.101)	(0.0751)	(0.101)	(0.0753)
Frequent gambler			−0.189	0.388^***^	−0.192	0.359^***^	−0.199	0.346^***^
			(0.175)	(0.109)	(0.175)	(0.110)	(0.175)	(0.110)
Myopic view of the future					0.213^*^	0.249^**^	0.202	0.227^**^
					(0.126)	(0.0969)	(0.126)	(0.0974)
Level of risk preference					0.0764	0.727^***^	0.0855	0.742^***^
					(0.248)	(0.185)	(0.249)	(0.186)
Current level of happiness							−0.545^**^	−0.988^***^
							(0.271)	(0.203)
Anxiety about health							−0.00764	−0.00506
							(0.0955)	(0.0722)
Constant	0.683	0.181	0.617	0.264	0.481	−0.456	0.325	−0.735
	(1.370)	(1.057)	(1.373)	(1.065)	(1.383)	(1.077)	(1.381)	(1.081)
Observations	3,706	3,706	3,706	3,706	3,706	3,706	3,706	3,706
Log likelihood	−2430	−2430	−2400	−2400	−2388	−2388	−2376	−2376
Chi2 statistics	495.7	495.7	542.8	542.8	560.3	560.3	568.4	568.4
*p*-value	0	0	0	0	0	0	0	0

Robust standard errors in parentheses,

*** p < 0.01,

** p < 0.05,

* *p < 0.1*.

## Discussion

As mentioned earlier, there is an important difference between financial literacy and financial education in the Japanese context. Financial literacy measures respondents' current level of financial knowledge and focuses on the investment perspective, whilst financial education focuses on the savings behavior. Our results demonstrate that both financial literacy and financial education have a significant impact on smoking behavior. Hence, the focus of our discussion here is the final model, as shown in Table 8.

Among Models 3.1-3.4, both financial literacy and financial education have a significantly negative impact on smoking behavior, except financial education in Model 3.2. These inverse relationships indicate that respondents with a high level of financial literacy and those who received financial education were less likely to be current smokers. The findings support our hypothesis that financially literate and financially educated people, who have the ability to make more rational decisions, are less likely to make the irrational decision to smoke. Watanapongvanich et al. (59) also found an inverse relationship between financial literacy (as a proxy for a rational decision-making tool) and gambling behavior (as a proxy for irrational decisions).

For the demographic variables, we find that being male, older (until a certain age), divorced, and having an education level below university degree, low household income, and low household assets are related to the current smoking status. Our results are consistent with those of previous studies, which found that smoking is more prevalent and progressive in males [e.g., (13, 73–78)] and persons in lower socioeconomic status (SES) [e.g., (73, 79–85)]. A plausible explanation is that persons in higher SES groups tend to have more knowledge about health risks and better access to health care resources and smoking cessation services (79, 84), which results in a lower rate of smoking prevalence compared to those in the lower SES group. In addition, persons with lower education levels may suffer from lower self-esteem compared to those who complete higher education and may be more likely to take up smoking as a self-enhancement mechanism (86). Regarding marital status, Lindström (87) and Pennanen et al. (81) found that smokers living without a spouse have higher daily smoking rates and nicotine dependence. Castrén et al. (88) explain that the dissolution of a marriage may cause people to undertake harmful activities.

For risky behaviors, respondents who exercise regularly are less likely to be current smokers, whilst respondents who are current drinkers and frequent gamblers are more likely to be current smokers. Prior studies also report a negative relationship between physical activity and smoking [e.g., (74, 89–91)]. We can predict these results because people who exercise regularly tend to be health conscious and avoid behaviors that cause health deterioration. In contrast, people who are already engaged in health-risk behaviors such as drinking alcohol and gambling tend to engage in other risky behaviors, including smoking. For example, Nichter et al. (92) argue that alcohol makes drinkers more comfortable with the experience of smoking and raises their smoking limit. Therefore, drinkers are more likely to smoke than non-drinkers [e.g., (93, 94)]. In addition, engaging in gambling also encourages smoking, especially during the game [e.g., (95–97)].

Our results related to a myopic view of the future and level of risk preference, which have a positive impact on smoking behavior, also support our findings on health-risk behaviors. Respondents who have a myopic view of the future will focus more on the present; in other words, they value the pleasant feelings from smoking today more than the adverse effects that smoking will have on their health in the future [e.g., (24, 25, 98)]. Furthermore, respondents with high levels of risk preference tend to engage in risky behaviors more than those with a low level of risk preference [e.g., (24, 99, 100)]. Therefore, respondents with a myopic view of the future and a high level of risk preference are more likely to be current smokers. Lastly, respondents who reported high self-rated levels of happiness are less likely to be current smokers. This finding is consistent with that of Chang et al. (101), who found that in Japan, France, and the UK, if people feel happier, they smoke less. As smoking gives smokers pleasure, they feel more relaxed and energetic; therefore, they use smoking as a coping mechanism to combat stress (101–103).

## Conclusion

In this study, we examined the relationship between financial literacy, financial education, and smoking behavior among the Japanese population. We hypothesized that financially literate and financially educated people who have the ability to make more rational decisions, are less likely to smoke. Using data from the PPS 2010, the probit regression results show that both financial literacy and financial education have a significantly negative impact on smoking behavior. In other words, a high level of financial literacy (which emphasizes knowledge of investments) and receiving financial education (which emphasizes savings behavior) significantly reduces the probability of being a current smoker. These inverse relationships indicate that rational decision-making ability reduces the tendency to engage in irrational behavior. In addition, we find that gender, age, education, marital status, household income and assets, risky behaviors, myopic view of the future, risk preference, and level of happiness also significantly predict the likelihood of a person being a current smoker.

Our findings suggest that promoting financial literacy and financial education could help mitigate smoking behavior. In the case of Japan, the government can integrate financial knowledge that focuses on the investment perspective into the current financial education program to enhance the impact of financial education and financial literacy as countermeasures to smoking. However, further research is needed to explore the impact of financial literacy and financial education as rational decision-making tools in terms of smoking and other health-related behaviors in different contexts.

However, this study has several limitations. First, we base our measurement of financial literacy on only three questions designed by Lusardi and Mitchell (63, 70). However, other studies also use this method, which makes financial literacy internationally comparable [e.g., (48, 59, 64–69, 71, 104, 105)]. Second, we define smoking behavior only in terms of participation in smoking, but not the amount of cigarette consumption or the level of tobacco dependence because of data unavailability. Despite these limitations, this study provides empirical evidence that suggests a means to enhance the rational decision-making ability of individuals through financial literacy and financial education to curtail smoking behavior.

## Data Availability Statement

The original contributions presented in the study are included in the article/Supplementary Material, further inquiries can be directed to the corresponding author/s.

## Ethics Statement

The studies involving human participants were reviewed and approved by Osaka University. The patients/participants provided their written informed consent to participate in this study.

## Author Contributions

YK: study design. SW, MK, and YK: analysis and interpretation of data. SW, PP, SO, and MK: writing of the report. All authors contributed to the article and approved the submitted version.

## Conflict of Interest

The authors declare that the research was conducted in the absence of any commercial or financial relationships that could be construed as a potential conflict of interest.
